# Early identification and rapid repositioning of a malpositioned infusion port catheter under real-time ultrasound guidance: A case report

**DOI:** 10.1097/MD.0000000000042126

**Published:** 2025-04-18

**Authors:** Lijing Guo, Zonghan Li, Bin Luo, Yizhou Bai

**Affiliations:** a Department of General Surgery, Beijing Tsinghua Changgung Hospital, School of Clinical Medicine, Tsinghua, China.

**Keywords:** catheter malposition, totally implantable venous access port, ultrasound

## Abstract

**Rationale:**

A totally implantable venous access port is a subcutaneously implantable, long-term infusion device first reported by Niederhuber in 1982.

**Patient concerns:**

totally implantable venous access port provides a reliable venous access route for patients requiring long-term infusion therapy and chemotherapy.

**Diagnoses:**

Due to its advantages of fewer complications, low infection rates, and ease of long-term use, it has become the primary choice for central venous access in breast cancer chemotherapy patients.

**Interventions:**

In 2021, both the American Society of Vascular Access and the European Society for Medical Oncology recommended the optimal position for the catheter tip to beat the cavoatrial junction, where the superior vena cava meets the right atrium. Similarly, domestic guidelines also recommend the cavoatrial junction as the ideal position for the catheter tip.

**Outcomes:**

Infusion port catheter malposition, where the catheter tip is located in vessels other than the superior vena cava, is a rare complication of port implantation. This can lead to infusion difficulties or port-related thrombosis.

**Lessons:**

Therefore, timely intraoperative identification of catheter malposition and effective adjustment is of significant clinical value.

## 1. Introduction

On October 10, 2023, our hospital performed a left chest wall infusion port implantation via left internal jugular vein puncture under real-time echocardiography guidance. During the surgery, the catheter was primarily malpositioned into the contralateral internal jugular vein. Using a cranial upright method, we quickly repositioned the catheter. The case is reported as follows.

## 2. Case introduction

The patient is a 53-year-old female who underwent a modified radical mastectomy for right-sided breast cancer atour hospital. Postoperative pathology indicated: right breast invasive carcinoma, grade II, score 7,IHC: ER (98%+), PR (98%+), HER-2 (2+), ki67 (60%), FISH test showed no amplification. Pathological staging: T2N1M0, molecular subtype: Luminal B. The MDT discussion planned for adjuvant chemotherapy post- surgery. The chemotherapy regimen included AC-T (doxorubicin + cyclophosphamide for 4 cycles, followed by docetaxel for 4 cycles). Before chemotherapy, the patient underwent a left chest wall infusion port (BARD Groshong, 8Fr) implantation under local anesthesia. The procedure involved puncturing the left internal jugular vein and guiding the catheter through the chest wall using real-time echocardiography. Intraoperative findings indicated catheter malposition, and postoperative X-ray showed the catheter had mistakenly entered the contralateral internal jugular vein. Under ultrasound guidance, the catheter was repositioned to the cavoatrial junction. The patient has since successfully completed the current cycle of adjuvant chemotherapy without any catheter-related complications or functional issues during the placement period.^[1-8]^

## 3. Infusion port surgical procedure

Trained general surgeons atour hospital follow the infusion port surgery guidelines for catheter placement. First, the insertion length of the infusion port catheter is estimated using a surface measurement method, measuring the distance from the puncture site to the left sternoclavicular joint plus 10 cm. For this patient, the intended insertion depth was 16 cm. The patient was positioned in the Trendelenburg position with her head turned to the right. During the procedure, the left internal jugular vein was punctured at the mid-point under ultrasound guidance, and the guidewire was inserted smoothly (ultrasound model: CHISON Q8). After exchanging the introducer sheath, the catheter was inserted without resistance, with blood aspiration and saline injection through a 10 mL syringe being smooth, and the patient experiencing no discomfort.

During the procedure, the ultrasound was switched to the transthoracic echocardiography mode (using a phased array probe). After covering the probe with a sterile sleeve, it was placed at the apex of the heart, pointing towards the right sternoclavicular joint. The probe position and direction were adjusted until a 4-chamber view of the heart was obtained. Since the catheter was not visualized in the right atrium, catheter tip malposition was suspected. The sheath was withdrawn, and the catheter length exposed at the skin surface was 16 cm. The catheter was connected to the injection port, the skin incision was sutured, and the area was bandaged. The patient reported no discomfort during the catheter placement.

**Figure 1.**
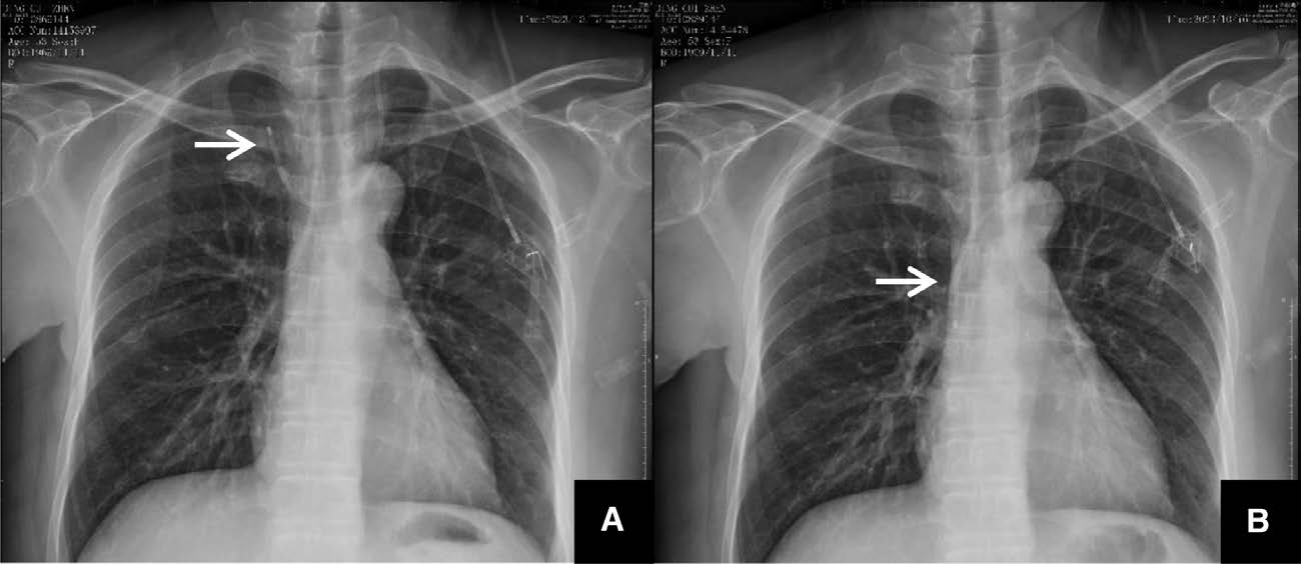
(A) Anteroposterior chest X-ray showing catheter tip malpositioned into the internal jugular vein. (B) Anteroposterior chest X-ray showing catheter tip repositioned.

A chest X-ray taken immediately after the procedure showed the catheter in a U-shape, with the tip located in the right neck, diagnosing the catheter as malpositioned into the contralateral internal jugular vein (see Fig. 1A). The patient was returned to the procedure room, where the puncture site in the left neck was re-sterilized and draped. The catheter was exposed and withdrawn approximately 10 cm, retracting it into the left brachiocephalic vein. At this point, the assistant helped the patient adjust her head from a right-turned to an upright position. The withdrawn catheter length was then slowly and completely reinserted. Ultrasound confirmed no catheter echo in the right internal jugular vein or subclavian vein. A follow-up chest X-ray indicated the catheter tip at the cavoatrial junction (Fig. 1B), at the level of the inferior edge of the T7 vertebral body, completing the adjustment of the infusion port catheter.

## 4. Discussion

Totally implantable venous access port is a commonly used vascular access in clinical practice, which can be implanted via various vascular puncture routes for long-term use. Common vascular puncture routes include the internal jugular vein, subclavian vein, basilic vein, and femoral vein. Catheter malposition occurs when the catheter tip, after infusion port implantation, is not in the superior vena cava but in other vessels or the heart, with an incidence rate of about 0.3% to 6%.^[9,10]^ Immediate intraoperative malposition is referred to as primary catheter malposition. Malposition locations include the aorta, contralateral brachiocephalic vein and subclavian vein, ipsilateral or contralateral internal jugular vein and its branches, azygos vein, right or left internal thoracic vein, pericardiophrenic vein, internal mammary vein, right atrium, and right ventricle. Compared to the subclavian vein puncture route, the internal jugular vein puncture route has a lower incidence of catheter malposition, about 0.2% to 1.4%.^[11,12]^ Primary catheter malposition mainly occurs on the left side, with the catheter tip often malpositioned into the bilateral brachiocephalic veins and the left internal thoracic vein.

Studies have shown that the malposition rate of the left internal jugular vein is 1.4%, while that of the right internal jugular vein is 0.25%.^[13]^ The higher malposition rate for the left internal jugular vein compared to the right is related to the relatively straight anatomical path from the right internal jugular vein to the superior vena cava.^[14]^The internal jugular vein originates at the base of the skull and descends, merging with the subclavian vein behind the sternoclavicular joint to form the brachiocephalic vein. The right brachiocephalic vein descends almost vertically, while the left brachiocephalic vein runs obliquely behind the upper part of the sternum, crossing in front of the aortic arch branches, and merges with the right brachiocephalic vein at the right first sternocostal junction to form the superior vena cava. The anatomical differences between the 2 sides’ vascular pathways are the main reason why primary catheter malposition is more likely to occur with left internal jugular vein access compared to right internal jugular vein access.

In this case, the patient, who had undergone a modified radical mastectomy for right-sided breast cancer, was to receive postoperative adjuvant chemotherapy, followed by radiotherapy. Therefore, the left internal jugular vein puncture route, opposite the surgical area, was chosen as the preferred venous access for the infusion port implantation. The catheter was initially malpositioned into the right internal jugular vein region. Currently, there are few literature reports on catheter malposition to the contralateral internal jugular vein following infusion port implantation via the internal jugular vein puncture route. Some studies suggest that if a catheter malposition occurs post- implantation and the patient has no symptoms, with smooth blood return and fluid infusion, the port can be used normally. However, it is essential to closely monitor the patient for any related complications during use.^[15]^

Catheter malposition can affect blood flow or the direction of infused fluids, leading to turbulence and increasing the risk of thrombosis. Additionally, the purpose of the infusion port implantation in this patient was to administer chemotherapy drugs. If the catheter malposition is not corrected, it could result in a transient increase in the concentration of chemotherapy drugs in the head, causing discomfort. Therefore, it is necessary to correct the catheter malposition.

Currently, there are 2 main approaches to managing infusion port catheter malposition: In previous studies,^[16,17]^ researchers often used snare devices, pigtail catheters, or a combination of both for interventional adjustments, with an overall success rate of up to 93%. However, the use of snare devices involves high material costs and a risk of vascular injury.^[18]^Another method involves reopening the original puncture site, withdrawing the infusion port catheter, and adjusting it under DSA (digital subtraction angiography). This can be done by injecting contrast agent through the injection port to perform angiography and real-time visualization of the catheter tip position. However, this method may require multiple attempts to place the catheter tip in the ideal position, potentially prolonging the surgery and increasing the risk of infection.

In this case, the possible cause of catheter malposition was the patient’s excessive rightward tilt of the neck during catheter placement, which created an excessive angle between the right internal jugular vein and the brachiocephalic vein. This likely caused the catheter to follow the puncture sheath path into the right brachiocephalic vein and then into the right internal jugular vein. Therefore, for this patient, the decision was made to reopen the original puncture site and adjust the catheter. Based on surface measurements, the catheter was withdrawn by approximately 10 cm until the tip retracted into the left brachiocephalic vein. Then, with the assistant’s help, the patient’s head was adjusted to a neutral position, reducing the angle between the right internal jugular vein and the brachiocephalic vein, allowing the catheter to be successfully repositioned.

Currently, to prevent intraoperative primary catheter malposition, techniques such as intracavitary electrocardiography (ECG) and X-ray fluoroscopy can be used to assist in locating the catheter tip during surgery. These methods help to promptly detect and dynamically adjust any malposition, thereby preventing primary catheter malposition.^[19]^ However, X-ray fluoroscopy requires specialized equipment and specific operating room radiation protection conditions. Repeated fluoroscopy can increase radiation exposure risk for both patients and medical staff. Intracavitary ECG is susceptible to interference from external electronic devices and is not suitable for patients with arrhythmias, atrial fibrillation, or implanted pacemakers, where ECG rhythms are abnormal.^[20]^

In this case, transthoracic echocardiography was used to locate the infusion port catheter tip, avoiding X-ray radiation and allowing real-time monitoring of the catheter position. During the procedure, the ultrasound probe was placed at the cardiac apex, and the catheter insertion length was adjusted under a 4-chamber view. If the catheter tip was not visible in the right atrium despite exceeding the premeasured insertion length, catheter malposition was suspected. Using the cranial upright method to adjust the catheter position, intraoperative ultrasound helped to promptly exclude catheter re- malposition into the internal jugular vein, thus avoiding repeated procedures and reducing the risk of infection.^[21]^

In summary, using real-time ultrasound guidance for infusion port implantation allows for early detection of primary malposition, and the cranial upright method facilitates quick and successful repositioning. This approach is simple and effective, preventing the need for repeated infusion port implantation surgeries or multiple adjustments, thereby reducing the patient’s psychological and financial burden. It is worth further promotion.

## Author contributions

**Conceptualization:** Lijing Guo.

**Data curation:** Lijing Guo.

**Formal analysis:** Zonghan Li.

**Investigation:** Bin Luo.

**Methodology:** Lijing Guo.

**Software:** Lijing Guo.

**Supervision:** Bin Luo.

**Project administration:** Zonghan Li.

**Validation:** Bin Luo, Yizhou Bai.

**Visualization:** Zonghan Li.

**Writing – original draft:** Lijing Guo.

**Writing – review & editing:** Bin Luo, Yizhou Bai.
